# COVID-19 Misinformation: A Potent Co-Factor in the COVID-19 Pandemic

**DOI:** 10.7759/cureus.30026

**Published:** 2022-10-07

**Authors:** Ishan Aiyer, Likhita Shaik, Rahul Kashyap, Salim Surani

**Affiliations:** 1 Education, Blair Academy, Blairstown, USA; 2 Family Medicine, Hennepin Healthcare, Minneapolis, USA; 3 Critical Care Medicine, Mayo Clinic, Rochester, USA; 4 Research, WellSpan Health, York, USA; 5 Anesthesiology, Mayo Clinic, Rochester, USA; 6 Pulmonary, Critical Medicine, and Pharmacology, Texas A&M University, College Station, USA; 7 Clinical Medicine, University of Houston, Houston, USA

**Keywords:** covid-19 retro, vaccine hesitancy, vaccine, ethics, infodemic, disinformation, misinformation, social media, covid-19

## Abstract

COVID-19, the biggest global health crisis of our times was complicated by an equally potent co-factor: the misinformation infodemic. A confluence of unique factors led to the emergence of the crisis of misinformation, including the widespread reach of social media, the lack of credible sources and strategies for information dissemination, and the sticky and virulent nature of the misinformation campaigns. One of the primary targets of the misinformation campaign was the COVID-19 vaccine effort, leading to significant impediments to implementing an effective and successful vaccination campaign. The time to act is now and will need a concerted multipronged approach with a close partnership between scientists, public health agencies, government agencies, and social media companies to foster accuracy in the exchange of health information in social media and curb the menace of misinformation. This paper aims to review the scope of the problem and examine strategies to help mitigate it.

## Introduction and background

The COVID-19 pandemic has been the worst global health crisis of our time, infecting an estimated 600 million people and causing 6 million deaths, and it continues to be a lingering threat worldwide [1]. What made this pandemic different from any other that humanity has faced in the past was not just about how potent and virulent the SARS-CoV-2 virus was, but also that it had an ally that was even more virulent and potent: the infodemic of misinformation. Despite glaringly clear evidence of vaccine and mask efficiency, and the global scientific effort that resulted in making an effective vaccine accessible all over the world in record time, misinformation related to masks and vaccines has made their global adaption a challenge. With the emergence of effective antivirals, people continued to get affected in large numbers largely due to the misinformation virus playing a crucial role in the pandemic. While there is controversy on whether the COVID-19 pandemic is becoming endemic or not, it is quite clear that the misinformation pandemic is well entrenched in our society even before the COVID-19 pandemic and has just exploded in the past few years, both regarding the spreading of COVID-19 misinformation as well as political issues. It is going to require a sustained global effort to mitigate its ongoing onslaught [1].

## Review

Misinformation is defined as "publicly available information that is misleading or deceptive relative to the best available scientific evidence, and that runs contrary to statements by actors or institutions who adhere to scientific principles" [2]. The term disinformation is used to refer to "deliberate, engineered falsehoods circulated with malicious intent or to serve a personal, political, or economic agenda." "Infodemic" is another term defined by the WHO as referring to "false and misleading information that causes confusion, risk-taking behaviors and mistrust of health officials" [2]. The problem becomes compounded since the majority of people now turn to online resources and social media for information about COVID. One study reported that 72% of Americans turned to online news and social media sources for COVID-19 information, with 47% reporting that the source was social media [3]. Cinelli et al., employing epidemic modeling for the dissemination of information, showed high measures of the “transmissibility” of posts on various social media platforms [4]. This misinformation pandemic led to an erosion of trust in science, and scientifically based expert guidelines, as well as in governmental interventions, and public health responses to COVID-19 [5].

Misinformation and vaccination

While misinformation has been impactful on a multitude of aspects related to COVID-19, including mask-wearing, use of chloroquine, and ivermectin, among others, the single biggest area where there has been a sustained and relentless misinformation campaign has been on the issue of vaccination; both in terms of efficacy and, much more so, in terms of its safety. Concerns about potential safety aspects particularly the side effects and the rapid pace at which these vaccines were developed are some of the primary reasons for COVID-19 vaccine hesitancy. The WHO has defined vaccine hesitancy as "the reluctance or refusal to vaccinate although the availability of vaccines" [6-7]. Vaccine hesitancy has been the primary culprit in derailing public health vaccination strategies globally and one of the primary drivers of misinformation on social media. The US Surgeon General identified misinformation as the “greatest threat to COVID-19 vaccination efforts” [8]. Table 1 illustrates common topics of COVID-19 misinformation.

**Table 1:** COVID-19 Misinformation

Covid-19 Misinformation
Mask
Vaccines-General
Vaccines Efficacy
Vaccines Side effects
Chloroquine
Ivermectin

**Figure 1:**
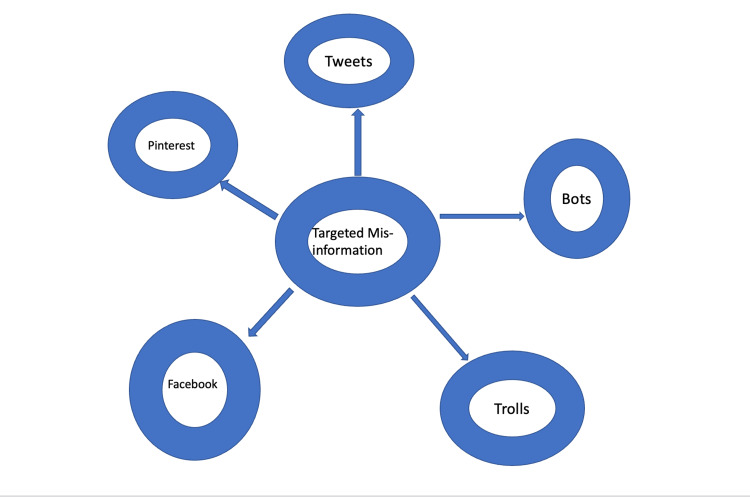
Targeted misinformation towards females, youths, lower-income populations, and disparity population

Muric et al. analyzed antivaccine-related tweets from Twitter’s application programming interface (API) and found that misinformation originated from websites with low and dubious credibility and that many accounts with antivaccination content were right-leaning politically [9]. Bots, short for software robots, are typically automated spam accounts and likely play a critical role in orchestrating the misinformation spread. One study found that up to 66% of bots are discussing COVID-19 [10]. Another potent source of misinformation is "trolls". This term is used for individuals who deliberately misrepresent their identity with the purpose of promoting discord. Amplification is a term used to describe a commonly used misinformation strategy by employing multiple bots and trolls to create impressions of false equivalence or consensus [11]. Similarly, another study [12] by Ferrara et al., found that automated bots tweeted significantly more COVID-19-related content as compared to non-bot accounts, and a content analysis of these tweets revealed that they significantly promoted political conspiracies and divisive content. Figure 1 illustrates the targeted misinformation.

Hughes et al. [13] employed qualitative coding methodology to analyze and identify specific patterns of content that were commonly employed by anti-vaccine media. A framework called "the 5C model of the drivers of vaccine hesitancy" provides five main individual-level determinants for vaccine hesitancy: confidence, complacency, convenience (or constraints), risk calculation, and collective responsibility [14]. A study from the United Kingdom (UK) found that demographic variables associated with COVID vaccine hesitancy included youth, female gender, and low income or education. These subjects also tended to rely heavily on social media for information and lacked trust in science [15]. Wilson et al. were able to demonstrate a significant relationship between social media use to organize offline action and concerns about vaccine safety, and they also found an influence from foreign disinformation campaigns [16].

Social media may help promote disinformation by making use of vivid imagery and content [17]. Betsch et al., describe that based on the “fuzz-trace theory,” where individuals process new information through verbatim memories which incorporate many precise details and gist memories that only contain a summarized bottom line meaning, they make decisions usually based on the gist memories rather than detail memories [18]. Content on social media that is vague and anecdotal tends to be shared more readily and is more likely to go viral, as opposed to evidence-based information that tends to be duller and more theoretical.

Machine learning-based models have been shown to be effective in detecting misinformation regarding COVID-19 vaccines on social media platforms. Bots and trolls are programmed to spread misinformation. Machine learning can help in detecting and mitigating this misinformation. Artificial intelligence can also utilize several machine learning models to classify fake news and tweets [19]. At least two studies have attempted to create datasets of tweets to annotate misinformation about covid vaccines - Covid Lies and Covax Lies [20,21]. As these detection systems are improvised and additional targets of misinformation are added, these efforts may result in developing misinformation inoculation interventions on social media platforms relevant to COVID-19 vaccination.

Strategies to combat the misinformation virus

There is an urgent need for interventions to mitigate the effects of this infodemic, but thus far, the best way to achieve effective solutions has been elusive.

Promoting Accuracy in Information

As the understanding of COVID evolves, separating valid information from misinformation becomes a moving target and hence challenging to actualize. The availability of abundant and unreliable sources of information poses additional challenges. The information chain needs structure, wherein information production needs to be limited and mainly resourced to standardized public health portals (e.g. World Health Organization, Center for Disease Prevention) as reliable sources of information [22]. Artificial intelligence and machine learning models can help in recognizing and mitigating the fake and false information either spread via the algorithm or the trend and help in flagging and mitigating it [19]. This available information should be relayed or promoted by leveraging mass media platforms like social media, television, newspapers, and radio. Implementing this structural differentiation between information source and relay of information is vital to minimize the spread of wrong content and ensure accurate data is available to end users [22]. Pennycook et al., in one study, showed that a simple accuracy reminder could help participants in truth discernment and influence their subsequent sharing behavior [23].

Clarifying Misinterpreted Ideas

Eradicating health-related misconceptions is the first key to promoting community health [24]. This can be established by individuals that can best present facts in non-scientific jargon, which for most populations is their trusted community health leaders, which includes physicians, researchers, mental health workers, community health workers, or social workers [25]. Bautista et al. proposed a two-phased conceptual model. An authentication phase as well as a correction phase, which healthcare professionals can use to approach correcting health misinformation on social media (e.g., Twitter and Facebook) [26]. Special campaigns that provide opportunities for them to interact with their patients to clarify doubts and misconceptions through emotive language and imagery can be helpful. This process can be accelerated by health agencies by fostering an increased social media presence that can be utilized in promoting such health events, such as legal consequences and financial penalties for spreading intentional false information either by an act of commission or omission.

Social Media Self-Regulation

Social media plays a big role in influencing the community. Healthcare providers can effectively disseminate useful information through social media platforms. Social media channels can play a proactive role in the continued propagation of pro-health ideas and/or curtailing anti-health ideas. All the major social media companies issued a joint statement on their efforts to minimize misinformation about COVID-19 on their platforms [26]. Pinterest redirected vaccine search results on their site to credible information sources such as the CDC. Facebook or Instagram reduced the ranking and rejected ads of groups and pages that promoted vaccine misinformation [27,28]. Social media can also act as barricades to identify potentially harmful misinformation preachers. Various social media platforms have partnered with government health agencies to link health-related information to official websites to help relay accurate information. 

Inoculation

As misinformation may prove resistant to correction, an alternative approach derived from inoculation theory is to proactively prepare people for potential misinformation by explaining the logical inconsistencies and pitfalls inherent in misleading communications beforehand. The rationale for this theory is that "inoculating" people in this manner will enable them to better perceive flaws in the arguments and information presented to them and thereby be able to identify them as being deceptive [29]. Just as how vaccines work, "inoculation" messages introduce counterarguments in people’s minds that can help generate resistance to future misinformation.

Law Enforcement and Community Movement

While social media companies in the United States are subject to US Federal Trade Commission regulations as any other US-based business, they are free of any oversight by the US Federal Communications Commission. More specifically, Section 230 of the Communications Act of 1934 absolves social media companies of an obligation to audit or censor user-generated content on their platforms by not considering them as publishers of information as other media entities. Hence the onus of curbing disinformation is largely a result of internal self-monitoring on the part of these companies, although there is evidence to show that social media platforms may, in many instances, be complicit in disseminating identifiable misinformation [30]. Health misinformation needs to be viewed as a public health crisis, and there is hence an urgent need for federal oversight and regulation of social media companies in the same manner as any other media source. Currently, Congress is actively looking into the security system and details for major companies as Facebook and Twitter. The company has stated that they have become more vigilant in recognizing the fake news and even blocking the accounts of users spreading the fake news [31-32].

The United Nation International Children's Emergency Fund (UNICEF) has created a field guide to target vaccine misinformation which provides a comprehensive structure organized into three phases: Listen, Understand, and Engage [33-36]. The phase “listen” refers to the creation of an active and dynamic social listening system that includes appropriate monitoring tools, including the use of “virality scores” and “rumor logs.” The “understand” phase proposes a Five Pillars of Verification system: provenance, source, date, location, and motivation for responding to information and creating a "risk evaluation matrix." The phase "engage" explores promoting social media health literacy and inoculation strategies.

## Conclusions

The infodemic is here to stay and has potentially more lethal ramifications than even the COVID-19 pandemic. Unlike the hugely successful vaccination development program for COVID-19, inoculation strategies for this infodemic are not as easy to resolve and involve a complex interplay between social media companies, medical professionals, researchers, implementation scientists, and federal agencies.
